# Mycobacteria Amplicon Sequencing Tool: automated resistance prediction and lineage classification for *Mycobacterium tuberculosis*

**DOI:** 10.1128/mra.00032-26

**Published:** 2026-04-27

**Authors:** Idowu B. Olawoye, Maxim Fedorov, Robert A. Petit, Jennifer L. Guthrie

**Affiliations:** 1 Department of Microbiology & Immunology, University of Western Ontario 6221, London, Ontario, Canada; 2 Wyoming Department of Health, Wyoming Public Health Laboratory, Cheyenne, Wyoming, USA; 3 Public Health Ontario 153300, Toronto, Ontario, Canada; University of Michigan, Ann Arbor, Michigan, USA

**Keywords:** bioinformatics, software, mycobacteria, antimicrobial resistance

## Abstract

The Mycobacteria Amplicon Sequencing Tool (https://github.com/guthrielab/MAST) is a modular Nextflow pipeline for antimicrobial resistance prediction and lineage classification of *Mycobacterium tuberculosis* from amplicon or whole-genome sequencing data sets. The workflow automates read processing, variant calling, and annotation to produce standardized, human-readable reports in DOCX and TSV format.

## ANNOUNCEMENT

*Mycobacterium tuberculosis,* the causative agent of tuberculosis, is responsible for an estimated 1.2 million deaths globally each year (1). Given its global burden and rising antimicrobial resistance (AMR), *M. tuberculosis* is routinely monitored through public health surveillance, with genome sequencing increasingly used to predict resistance and characterize circulating lineages. These data provide insight into phylogeographic distribution, transmissibility, and resistance patterns (2–4). In addition, targeted amplicon sequencing has recently been used to rapidly detect resistance-associated variants (5).

To support public health laboratories and researchers conducting targeted amplicon sequencing of *M. tuberculosis*, we have developed Mycobacteria Amplicon Sequencing Tool (MAST), a Nextflow-based pipeline that parallelizes the analysis of whole-genome and targeted amplicon sequencing data (6). The pipeline detects sequence variants and compares them against a catalog of 11,390 high-confidence variants and their associated drug-resistance phenotypes curated by the World Health Organization (2nd edition) (7, 8). It also applies a lineage barcode schema to assign isolates to 24 major lineages and sub-lineages, enabling standardized lineage identification alongside AMR prediction (3).

**Fig 1:**
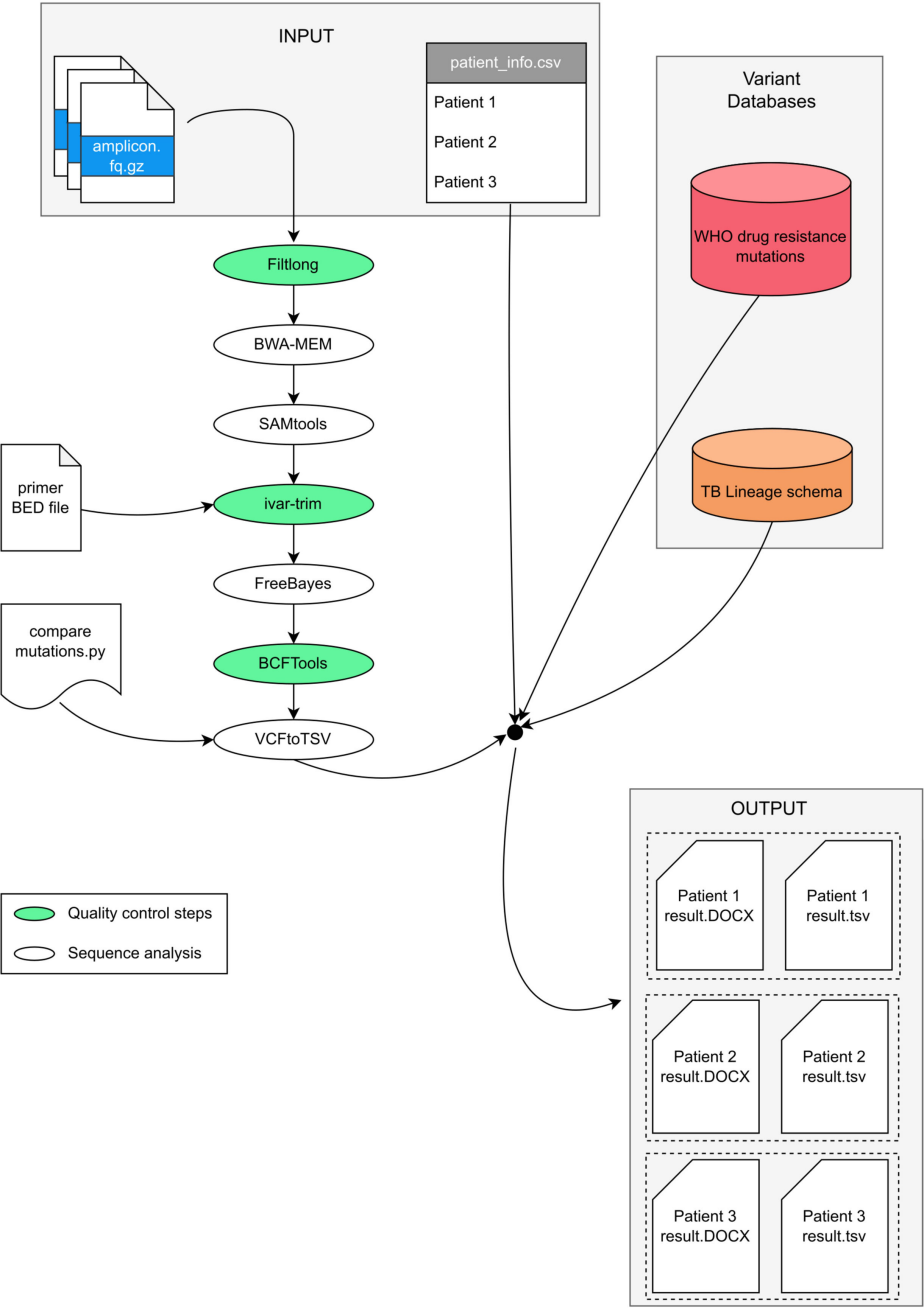
Schematic overview of the MAST workflow.

MAST is an open-source bioinformatics pipeline for the analysis of *M. tuberculosis* amplicon sequencing data and is available on GitHub. It uses conda dependencies such as Filtlong (9) for quality trimming, BWA-MEM (10), Pysam (11), and SAMtools (12) for read alignment and BAM manipulation, Freebayes (13) for variant calling, and a custom Python script to compare mutations against the drug-resistance and lineage databases as seen in Fig. 1. Acceptable input files include one or multiple single-end FASTQ files (Illumina or Nanopore sequence data) and a CSV file containing patient information; these inputs generate two output files:

1. A user-friendly MS Word (.docx) report summarizing the predicted drug-resistance profile, lineage assignment, and selected patient information provided by the user.
2. A tab-delimited summary file (.tsv) suitable for downstream parsing, integrating with other tools, or storage in a database.

We evaluated MAST against the widely used tool TB-Profiler (14) version 6.6.5, using 168 publicly available *M. tuberculosis* genomes selected from NCBI, restricting to genomes with an average sequencing depth of at least 30× and including isolates spanning major global lineages with both drug-susceptible and resistant profiles. Lineage predictions and AMR outputs produced by MAST were compared with TB-Profiler using default parameters. Lineage assignments were identical for all genomes (100% concordance). AMR predictions showed 99.7% concordance, with 350 of 351 drug-specific resistance calls matching those reported by TB-Profiler. The single discordant call corresponded to a variant that fell below MAST’s reporting threshold due to filtering criteria, reflecting a difference in variant-handling logic rather than an incomplete mutation list (Table 1).

**TABLE 1:** Comparison of TB-Profiler and MAST demonstrating concordance in lineage assignment and antimicrobial resistance prediction

Category	MAST	TB-Profiler	Concordance %
Lineage assignment	168	168	100
AMR prediction	350	351	99.7

A key advantage of MAST is its ability to process both targeted amplicon and whole-genome sequencing data, while remaining accessible to users with diverse levels of bioinformatics expertise. To support reproducibility and practical utility, we engaged domain experts to review the workflow and its applicability in routine settings. The pipeline will be maintained, supported, and openly available to the community.

## Data Availability

MAST is available on GitHub under the GNU General Public License version 3.0 at https://github.com/guthrielab/MAST. An example data set to test the pipeline is available on Zenodo (DOI: https://doi.org/10.5281/zenodo.19041451). The workflow itself is archived on Zenodo and accessible via DOI: https://doi.org/10.5281/zenodo.19166350.

## EDITOR'S NOTE

**Editor's. Note:** This article was handled by Pat Schloss, who acted as a Guest Editor in consultation with the Editor in Chief, Irene Newton. Dr. Schloss's role as the Guest Editor of this article is outside of his duties as the chair of the Journals Committee.
